# Supplementary material for: Spread Layers of Lysozyme Microgel at Liquid Surface
Source: Polymers (Basel). 2022 Sep 23;14(19):3979. doi: 10.3390/polym14193979 (PMC9570608; doi:10.3390/polym14193979)
Supplement: Supplementary file 1 [file polymers-14-03979-s001.zip › SUPPORTING INFORMATION LYS SPH_2.pdf]

SUPPORTING INFORMATION

Spread Layers of Lysozyme Microgel at Liquid Surface

Olga Yu. Milyaeva[1], Alexander V. Akentiev[1], Alexey G. Bykov[1], Shi-Yow Lin[2], Giuseppe Loglio[3], Reinhard Miller[4], Alexander V. Michailov[1], Ksenia Yu. Rotanova[1], Boris A. Noskov[1*]

[1] Department of Colloid Chemistry, St. Petersburg State University, St. Petersburg, Russia
[2] National Taiwan University of Science and Technology, Chemical Engineering Department, Taipei, Taiwan
[3] Institute of Condensed Matter Chemistry and Technologies for Energy, Genoa, Italy
[4] Technical University Darmstadt, Darmstadt, Germany
* Correspondence: borisanno@rambler.ru

Abstract: The spread layers of lysozyme (LYS) microgel particles were studied by surface dilational rheology, infrared reflection-absorption spectra, Brewster angle microscopy, atomic force microscopy and scanning electron microscopy. It is shown that the properties of LYS microgel layers differ significantly from those of ß-lactoglobulin (BLG) microgel layers. In the latter case the spread protein layer is mainly a monolayer and the interactions between particles lead to the increase of the dynamic surface elasticity up to 140 mN/m. In contrast, the dynamic elasticity of LYS microgel layer does not exceed the values for pure protein layers. The compression isotherms also do not exhibit specific features of the layer collapse which are characteristic for the layers of BLG aggregates. LYS aggregates form trough three-dimensional clusters directly during the spreading process and protein spherulites do not spread further along the interface. As a result, the liquid surface contains large almost empty regions and some patches of high local concentration of the microgel particles.

Keywords: β-lactoglobulin, lysozyme, microgel particles, spread layers, IRRAS, BAM, AFM, SEM, surface dilational visco-elasticity

Corresponding Author

*E-mail: borisanno@rambler.ru

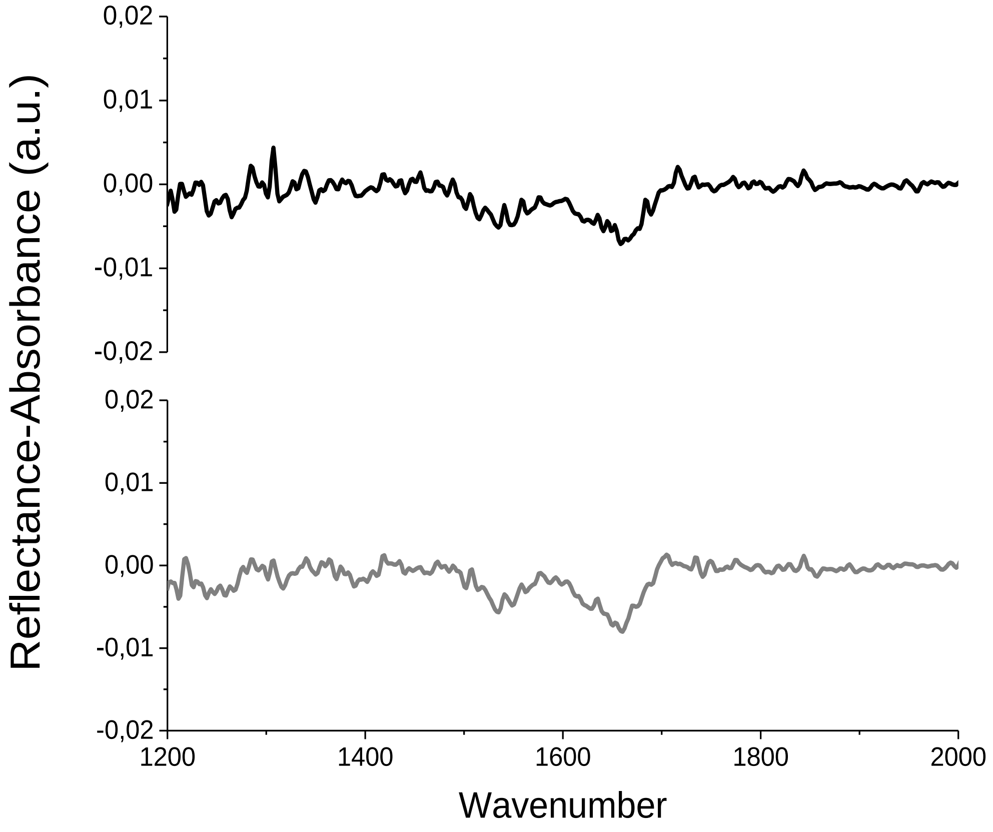

Figure S1. IRRAS spectra of spread layers of LYS microgel on water. The black line corresponds to the beam reflection from the surface region far from that where the dispersion was spread and the grey line corresponds to the beam reflection from the surface region where the spreading occurred.

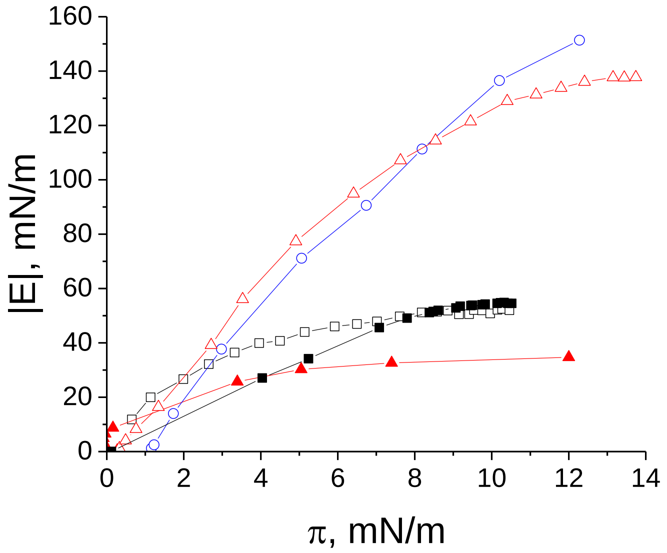

Figure S2. The dependences of the dynamic surface elasticity modulus on surface pressure of spread LYS (open symbols) and BLG (close symbols) layers on 0.1 M NaCl solution, pH=10 for LYS and pH=5.5 for BLG. The dispersion contains native protein (black squares), microgel particles (red triangels), microgel particles with addition of EtOH (blue circles).

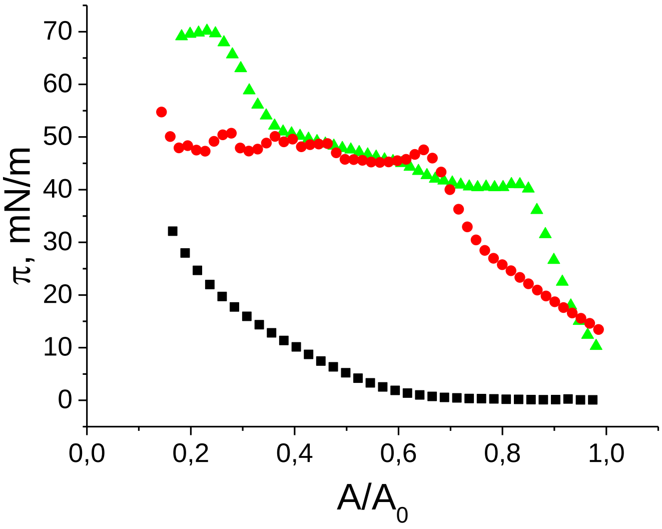

Figure S3. Compression isotherms of spread LYS microgel layers on phosphate buffer at pH=7 (black squares), LYS microgel with addition of EtOH layers on phosphate buffer at pH=7 (green triangles) and BLG microgel layers on 0.1 M NaCl water solution at pH=5.5 (red circles). Protein microgels was purificated with 3 steps.
